# Hair Mercury Levels Detection in Fishermen from Sicily (Italy) by ICP-MS Method after Microwave-Assisted Digestion

**DOI:** 10.1155/2016/5408014

**Published:** 2016-04-05

**Authors:** Giuseppe Giangrosso, Gaetano Cammilleri, Andrea Macaluso, Antonio Vella, Nicolantonio D'Orazio, Stefania Graci, Gianluigi Maria Lo Dico, Fabio Galvano, Margherita Giangrosso, Vincenzo Ferrantelli

**Affiliations:** ^1^Istituto Zooprofilattico Sperimentale della Sicilia “A. Mirri”, Via Gino Marinuzzi 3, 90129 Palermo, Italy; ^2^Dipartimento di Scienze Mediche, Orali e Biotecnologiche, Università degli Studi di Chieti, 66100 Chieti, Italy; ^3^Dipartimento di Scienze del Farmaco, Università degli Studi di Catania, 95125 Catania, Italy; ^4^Facoltà di Medicina e Chirurgia, Università degli Studi di Palermo, 90127 Palermo, Italy

## Abstract

A number of ninety-six hair samples from Sicilian fishermen were examined for total mercury detection by an Inductively Coupled Plasma Mass Spectrometry (ICP-MS) method. The mercury levels obtained were compared with mercury levels of 96 hair samples from a control group, in order to assess potential exposure to heavy metals of Sicilian fishermen due to fish consumption and closeness to industrial activities. Furthermore, the mercury levels obtained from hair samples were sorted by sampling area in order to verify the possible risks linked to the different locations. The overall mean concentration in the hair of the population of fishermen was 6.45 ± 7.03 *μ*g g^−1^, with a highest value in a fisherman of Sciacca (16.48 *μ*g g^−1^). Hair mercury concentration in fishermen group was significantly higher than in control group (*p* < 0.01). There was no significant difference in hair total mercury concentrations between sampling areas (*p* > 0.05). The results of this study indicate a greater risk of exposure to mercury in Sicilian fishermen, in comparison to the control population, due to the high consumption of fish and the close relationship with sources of exposure (ports, dumps, etc.).

## 1. Introduction

Pollution by heavy metals in water and soil increased considerably as a result of anthropogenic activities such as combustion of fossil hydrocarbons, mining, use of fertilisers, and urban waste. There is an increasing number of studies on the determination and quantification of these toxic elements in different species of the aquatic fauna. Heavy metals, in aquatic ecosystems, are more bioavailable and bioaccumulate in aquatic food chains to reach the highest concentration in the upper trophic levels [1, 2]. Mercury (Hg) is a toxic pollutant, which is distributed throughout the world from both natural and anthropogenic sources, and cannot be broken down in the environment. Fish food constitutes one of the routes of Hg uptake for humans [3–5] and dietary exposure to mercury products has fetotoxic, neurotoxic, and carcinogenic effects. Organic and inorganic mercury compounds have high affinity for sulfhydryl groups, can inhibit a large number of enzymes, can precipitate protein, and can kill every kind of living cell [6]. The level of mercury in humans can be determined by using human tissues as biopsy material [7]. Hair is easily available and gives significant information about element levels in the body as compared to other biopsy materials [8, 9]. This simple and noninvasive material is useful in identifying human populations in the environment at risk of heavy metals pollution [10, 11]. Furthermore, many minor and trace elements can be determined in such materials with good precision and sensitivity by a variety of analytical techniques [12, 13]. The concentration of heavy metals in hair can be linked to parameters characterizing and conditioning human life such as nutrition and work environment. The aim of this study was to report the estimated Hg exposure of fishermen of Sicily compared with a control group of the same sampling areas. The body burden of Hg exposure was assessed through the hair total Hg concentrations detected by an Inductively Coupled Plasma Mass Spectrometry (ICP-MS) method after microwave-assisted digestion.

## 2. Materials and Methods

### 2.1. Sampling Plan

**Figure 1:**
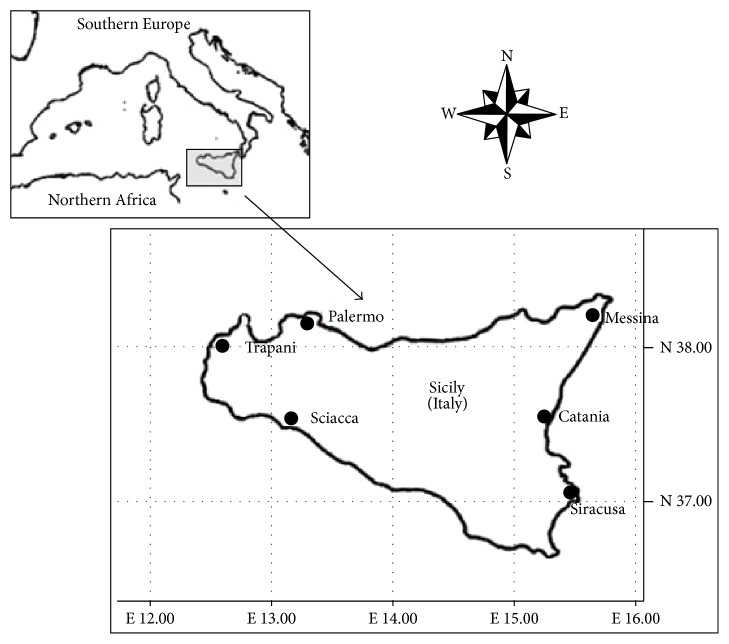
Sampling areas.

Ninety-six fishermen from Sicily with no dental problems were enlisted for hair sampling. Ninety-six additional samples were taken from a control group working in no maritime sectors. The control group was also selected to be of Italian nationality. They were young and healthy and none of them had dental problems. They are also occasional smokers, not exceeding five cigarettes day^−1^. Subjects were between 35 and 45 years of age and came from six areas of Sicily: Siracusa, Messina, Trapani, Sciacca, Catania, and Palermo (Figure 1). All the hair samples examined in this study (fishermen and control group) came from these six sampling areas. A questionnaire was completed for each volunteer (all were men) in order to assess his dietary habits. The per capita fish consumption of the control group ranged from 1 to 2 days/week, while the per capita fish consumption of fishermen ranged from 5 to 7 days/week. The two groups of volunteers had an average age of about 43 years and an average height of 1.72 m. About 5 g of head hair samples was collected using sterilized plastic scissor and washed with ultrapure water for total Hg levels detection by ICP-MS method.

### 2.2. Extraction of Samples

About 1 g of each sample of hair was weighed for the extraction. The extraction was performed with the use of PTFE microwave vessels with 2 mL of HNO_3_ (65% ultrapure) and 5 mL of ultrapure water. Subsequently, the vessels were closed and placed in a Multiwave digestion 3000 (Anton Paar) for digestion process. The digestion conditions are reported in Table 1.

After digestion, the content was transferred into 50 mL PVC test tubes. Vessels were washed twice with 10 mL of ultrapure water; subsequently, the content was transferred in a 50 mL test tube and diluted with ultrapure water for the ICP-MS analysis.

### 2.3. ICP-MS Analysis

The analysis was carried out in a ICP-MS Agilent 7700 series system (Agilent Technologies, California, USA) on the basis of two calibration curves, constructed by linear interpolation of at least 5 points, corresponding to the readings of standard solutions (Randon, Pennsylvania, USA) and the calibration blank, admitting a maximum error of 5% on the standard reading and a correlation coefficient *r* ^2^ > 0.98. Furthermore, an internal standard was added, in order to verify the instrument stability and quantify possible “matrix effects.” The concentration of the analytes was calculated by the following formula:

(1) $$\begin{array}{c} Cc=\frac{Cs\times Vf\times Vd}{1000\times p}, \end{array}$$

where *C*_*s*_ is the concentration of the analyte in solution calculated in the linear fit; *V*_*f*_ is the final volume (mL) of the sample solution; *V*_*d*_ is the dilution factor; *p* is the sample weight (g).

The instrument limit of detection (LoD = 0.01 *μ*g g^−1^) was calculated as the concentration associated with 3.3 times the standard deviation of the background noise recorded on seven measurements of the procedural blank.

### 2.4. Recovery Study

Trueness of the method was assessed by using the recovery of three concentration levels. All the samples were spiked with the right amount of concentration according to the scheme of Table 2, digested according to the preestablished microwave program, and then analyzed. The recovery was calculated as follows:

(2) $$\begin{array}{c} \text{recovery}\%=\frac{100C}{\text{spiked concentration}}, \end{array}$$

where *C* is the element concentration found. An acceptance limit between 90 and 100% was selected.

### 2.5. Data Collection and Statistical Analysis

Results obtained by chemical analysis were subdivided into fishermen hair samples (*n* = 96) and control hair samples (*n* = 96), for a total of 192 examined samples. The conditions of normal distribution and homogeneity of variance of the data have not been met; therefore a Wilcoxon rank sum test was carried out to evaluate significant differences between mercury hair levels of fishermen and control group. A Kruskal-Wallis test was carried out in order to assess significant differences in hair mercury concentrations between sampling areas. All the statistical tests were conducted by R®3.0.3. software.

## 3. Results and Discussion

**Figure 2:**
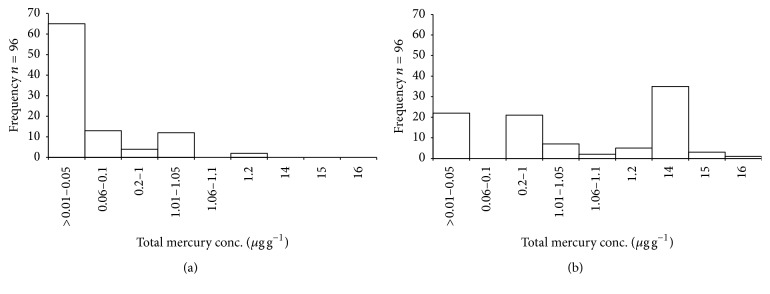
Histograms of the total Hg level distribution within the hair of fishermen (b) and the control population (a).

**Figure 3:**
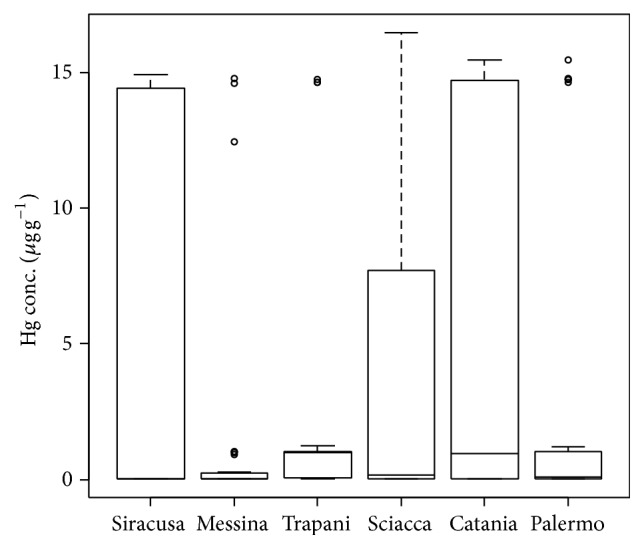
Total hair Hg data distributions according to sampling areas.

The recovery of total Hg was 98.9% for the three concentration levels. All the examined hair samples reached a total Hg concentration over the LOD. The hair mercury concentration distributions of fishermen and control populations are shown in Figure 2. The data obtained did not follow a log-normal distribution. About 40% of the fishermen's samples exhibited total Hg concentrations within the range of 14–16 *μ*g g^−1^, with a maximum value on a sample from a fisherman of Sciacca (16.48 *μ*g g^−1^), whereas for the control group about 68% of the samples were within the range of 0.01–0.05 *μ*g g^−1^. The overall mean concentration in the hair of the fishermen was 6.45 ± 7.03 *μ*g g^−1^. The overall mean concentration in the control group was 0.23 ± 0.4 *μ*g g^−1^. Wilcoxon test confirmed that the total hair Hg concentrations of fishermen were significantly higher than control group (*W* = 1592; *p* < 0.01, Figure 1). Those who ate fish more than five times a week had significantly higher hair mercury concentrations than those who ate fish less than three times a week. The results suggest high fish consumption and marine environment to be the major pathways of mercury into people's bodies. The hair Hg concentrations distribution according to sampling areas is shown in Figure 3. The lowest mean concentration was obtained in hair samples of Messina while the highest was obtained in samples from Siracusa (Table 3). The Kruskal-Wallis test revealed no significant differences between sampling areas (Kruskal-Wallis chi-squared = 7.2, df = 5, *p* > 0.05), suggesting that the following results do not depend on the environmental conditions of the different areas. However, hair samples that came from the southeast side of Sicily, where the density of industrial activities is greater (Syracusan petrochemical pole, Sciacca chemical pole, etc.), revealed the highest Hg concentrations. The average concentration of total Hg in fishermen of this study was much lower than the Portuguese fishermen of Madeira (39.76 *μ*g g^−1^ [14]); this large difference may be related to the different typologies of fishes consumed by the two fishermen populations. The most common fish consumed by Madeiran fishermen (*Aphanopus carbo*) have very high content of total mercury (0.90 ± 0.27 *μ*g g^−1^ [15]); on the contrary, recent study by Naccari et al. [16] has detected levels of total mercury in Mediterranean fishes between 0.132 ± 0.108 and 0.458 ± 0.059 *μ*g g^−1^ reducing the risk of exposure to heavy metals to fishermen. Indeed, the results obtained from this study were very close to the mean Hg concentration in the hair of fishermen from Aeolian Archipelago (southern Tyrrhenian Sea; 5.64 ± 3.73 *μ*g g^−1^ [17]) suggesting a strong correlation between type of fish consumed and mercury levels in the hair. All the fishermen investigated in this study have declared the consumption of Mediterranean mussel* Mytilus galloprovincialis. M. galloprovincialis *is recognised as one of the major Hg accumulators in Mediterranean ecosystems, probably due to its ecology and feed strategy [18–20]. For this reason* Mytilus galloprovincialis *can be one of the principal routes of Hg uptake for humans. Unfortunately, there are very few studies on the Hg levels assessment of seawater from south Mediterranean Sea. Bagnato et al. [21] have verified the distribution and evasion flux of mercury at the atmosphere/sea interface in the Augusta basin (SE Sicily, southern Italy), detecting that the intense industrial activity of the past has led to a high Hg pollution in the bottom sediments of the basin.

## 4. Conclusions

Results obtained confirmed hair samples as satisfactory biopsy materials for heavy metals determination. It is known that heavy metals penetrate in the brain as quickly as in the root of the hair [22]. Furthermore, a positive correlation between total mercury concentrations in hair and blood has been found [6] justifying the use of hair as an indicator of the exposure of the body to mercury [23]. Fishermen select and eat at their own risk a lot of large fish with their high total mercury and methylmercury load [24]. The exposure of this population to xenobiotic agents through the food and environment is one of the biggest concerns for health care institutions; for this purpose further studies are needed to have a full risk assessment on Italian population.

## Figures and Tables

**Table 1:** Digestion conditions.

Ph (Phase)	Power (Watts)	Ramp (minutes)	Hold (minutes)	Fan

1	450	10:00	40:00	1
2	0	0	15:00	3

**Table 2:** Concentration levels for the total Hg recovery study (10 replicates for level).

Level 1 (*µ*g g^−1^)	Level 2 (*µ*g g^−1^)	Level 3 (*µ*g g^−1^)
0.05	0.1	5

**Table 3:** Mean ± SD hair mercury concentration broken down by sampling areas.

Sampling area	Total Hg (mean ± SD *µ*g g^−1^)
Siracusa	4.71 ± 6.79
Messina	1.51 ± 4.16
Trapani	1.93 ± 4.11
Sciacca	3.94 ± 6.38
Catania	4.5 ± 6.85
Palermo	3.01 ± 5.79
